# QT dispersion in patients with systemic lupus erythematosus: the impact of disease activity

**DOI:** 10.1186/1471-2261-12-11

**Published:** 2012-02-27

**Authors:** Javad Kojuri, Mohammad ali Nazarinia, Mohammad Ghahartars, Yadollah Mahmoody, Gholam reza Rezaian, Lida Liaghat

**Affiliations:** 1 Cardiology Department, Shiraz University of Medical Sciences, Shiraz, Iran; 2 Internal Medicine Department, Rheumatology Group, Shiraz University of Medical Sciences, Shiraz, Iran; 3 Medical education, Cardiologist, Interventionis,t Cardiology Department, Namazi Hospital, Zand St., Shiraz, Iran

**Keywords:** SLE, Disease activity score, QT dispersion

## Abstract

**Background:**

Patients with systemic lupus erythematosus (SLE) have increased cardiovascular morbidity and mortality. Although autopsy studies have documented that the heart is affected in most SLE patients, clinical manifestations occur in less than 10%. QT dispersion is a new parameter that can be used to assess homogeneity of cardiac repolarization and autonomic function. We compared the increase in QT dispersion in SLE patients with high disease activity and mild or moderate disease activity.

**Methods and Results:**

One hundred twenty-four patients with SLE were enrolled in the study. Complete history and physical exam, ECG, echocardiography, exercise test and SLE disease activity index (SLEDAI) were recorded. Twenty patients were excluded on the basis of our exclusion criteria. The patients were divided to two groups based on SLEDAI: 54 in the high-score group (SLEDAI > 10) and 50 in the low-score group (SLEDAI < 10).

QT dispersion was significantly higher in high-score group (58.31 ± 18.66 vs. 47.90 ± 17.41 respectively; *P *< 0.004). QT dispersion was not significantly higher in patients who had received hydroxychloroquine (54.17 ± 19.36 vs. 50.82 ± 15.96, *P *= 0.45) or corticosteroids (53.58 ± 19.16 vs. 50.40 + 11.59, *P *= 0.47). There was a statistically significant correlation between abnormal echocardiographic findings (abnormalities of pericardial effusion, pericarditis, pulmonary hypertension and Libman-Sacks endocarditis) and SLEADI (*P *< 0.004).

**Conclusions:**

QT dispersion can be a useful, simple noninvasive method for the early detection of cardiac involvement in SLE patients with active disease. Concerning high chance of cardiac involvement, cardiovascular evaluation for every SLE patient with a SLEDAI higher than 10 may be recommended.

**Trial registration:**

Clinicaltrial.gov registration NCT01031797

## Background

Systemic lupus erythematosus (SLE) is a connective tissue disease characterized by the formation of autoantibodies and immune complexes. Patients with SLE have increased morbidity and mortality due to atherosclerotic cardiovascular disease. In SLE patients, as in diabetic patients, vascular complications may occur at a younger age than in the general population [1]. Moreover, once they occur, mortality may be about twice as high as in the general population [2]. In three cohort studies [3-5] cardiovascular mortality tended to cluster later in the course of disease, and was equal to and possibly even greater than that due to active lupus [6].

Cardiovascular involvement is now considered the third leading cause of death among patients with SLE. Autopsy studies have documented that the heart is affected in most patients [7]. Pericarditis and premature coronary atherosclerosis are the most prevalent cardiac manifestations [8]. Other less frequent associations include myocarditis, coronary arteritis, Libman-Sacks endocarditis, constrictive pericarditis, cardiomyopathy, congenital heart block and rhythm abnormalities. However, clinical manifestations occur in less than 10% of SLE patients [7,9].

QT-interval parameters, in particular heart rate-corrected QT interval duration, are presumed markers of increased cardiovascular risk and provide important prognostic information in clinical practice [10-14]. QT dispersion (QTd) is defined as the inter-lead variability in the duration of the QT interval in the 12-lead electrocardiogram (ECG). This new parameter can be used to assess the homogeneity of cardiac repolarization and autonomic function [15]. Increased heterogeneity of repolarization was shown in several heart diseases, and has been associated with increased risk of ventricular tachyarrhythmias [16]. Increased QTd has also been shown in patients with rheumatoid arthritis, ankylosing spondylitis and SLE [17-20].

The SLE disease activity index (SLEDAI) has been suggested as the best means to determine disease activity [21]. An SLEDAI > 10 is indicative of high disease activity, whereas SLEDAI equal to or below 10 indicates mild to moderate disease activity [22]. To the best of our knowledge there have been no studies that investigated the impact of SLEDAI on QTd. We therefore designed the present study to compare QTd in patients with SLE without overt cardiac involvement.

## Methods

Between October 2008 and March 2009, 124 patients older than 18 years with clinical evidence of SLE according to the revised criteria of the American College of Rheumatology [23] and with disease duration of one year or longer were selected from patients who were followed at the rheumatology outpatient clinic at Namazi Hospital in Shiraz, Iran. All patients gave written informed consent to take part in the study, which was approved by the local ethics committee. All patients fulfilled all of the following inclusion criteria: (1) no administration of drugs that would potentially influence QT duration except hydroxychloroquine, (2) no history of ischemic heart disease, congestive heart failure, atrial fibrillation, bundle branch block or abnormal serum electrolytes, (3) normal resting ECG and (4) a good-quality ECG recording to measure the QT interval. The exclusion criteria were: (1) moderate or severe valve disease, (2) atrial fibrillation and other ECG abnormalities, (3) systolic left ventricular dysfunction (ejection fraction < 50% or left ventricular end diastolic dimension > 5.5 mm), (4) unreliable identification of the end of the T wave in the ECG and (5) known presence of cardiac disease including hypertension, diabetes or coronary artery disease. Complete history, physical examination, ECG, and transthoracic echocardiography were recorded in all patients. Twenty patients with lupus were excluded from the study on the basis of our exclusion criteria.

The patients were divided in two groups based on modified SLEDAI (SLEDAI-2 K) scores equal to or less than 10 (mild to moderate disease activity) or above 10 (high disease activity). The treadmill exercise test with the Bruce protocol was used to rule out coronary artery disease. All patients were examined and their medical records reviewed to obtain clinical and serologic data and to calculate the SLEDAI score. Demographic variables (age, sex, weight and height), disease duration, the presence of atherosclerotic risk factors (smoking, hypertension, dyslipidemia, diabetes, obesity and family history) and medications in current use were recorded. Blood and 24-hour urine samples were obtained and the following laboratory tests were done: plasma glucose, serum total cholesterol, LDL, HDL, triglycerides, blood cell count, ESR, CRP, anti-dsDNA antibody, antinuclear antibody, serum complement and urine analysis.

Standard resting 12-lead ECGs were recorded in all patients with the same equipment and response frequencies at 25 mm/s paper-speed and 10 mm/mV amplitude. QT intervals were measured in each ECG lead from the onset of the QRS complex to the end of the T wave, defined as a return to the T-P baseline. If U waves were present, the patient were excluded from the study. Three consecutive cycles in each of the 12 leads were measured. All measurements were made by two experienced cardiologists who were blind to the patient's clinical status. QT-interval dispersion was defined as the difference between maximum and minimum non-rate-corrected QT-interval duration [24]. In this study corrected QTd was not calculated because previous studies showed that rate correction of parameters of repolarization dispersion is probably unnecessary and may even distort the values and predictive usefulness of QTd [25,26].

Transthoracic echocardiographic examination was done in all patients with a Zimence cardiac ultrasound scanner and 2.5-3.5 MHz transducers. Color Doppler, continuous and pulsed Doppler studies were done to evaluate left ventricular and valvular function. The following parameters were recorded by a cardiologist who was blind to the participants' clinical data: ejection fraction (EF), end systolic volume (ESV), ejection time (ET), isovolemic relaxation time (IVRT), isovolemic contraction time (IVCT), left ventricular mass (LV mass), tissue Doppler mitral valve parameters (A wave, S wave and E wave) and myocardial performance index (Tei index).

All statistical analyses were done with SPSS version 15.0 software. Continuous data are reported as means and standard deviations. Because the data for QTd were distributed normally, parametric tests were used to evaluate differences between the study groups. Means were compared by analysis of variance. Spearman's correlation rank test was used to find correlations among parameters. A P value below 0.05 was considered statistically significant. Trial was registered and approved by ethical committee of research faculty of Shiraz University of Medical Sciences.

## Results

We studied 50 patients with a SLEDAI equal to or below 10 (low score) and 54 patients with an SLEDAI > 10 (high score). The baseline characteristics of two groups of the patients are shown in Table 1. There were no significant differences with respect to age, systolic and diastolic blood pressure, hyperlipidemia, smoking, family history, disease duration, SLEDAI score, medications or body mass index. All patients in both groups had normal ejection fraction (65.83 ± 9.75 vs. 64.32 ± 11.15, respectively). None of the participants had significant valvular heart disease or other cardiac complaints. Treadmill exercise test results were negative in all patients.

**Table 1:** Baseline characteristics of two groups of SLE patients with high score and low score

Variable	High score group (n = 54)	Low score group (n = 50)
**Age (years)**	33.88 ± 11.34	35.32 ± 10.8

**Sex (Male:Female)**	3: 51	6: 44

**BMI(kg/m2)**	23.36 ± 4.69	24 ± 4.34

**Systolic blood pressure(mmhg)**	116.48 ± 12.76	117.60 ± 9.38

**Diastolic blood pressure(mmhg)**	77.87 ± 8.4	79.20 ± 5.2

**Duration of disease(year)**	6.64 ± 3.96	5.2 ± 3.87

**Left ventricular ejection fraction**	64.32 ± 11.15	65.83 ± 9.75

**Hydroxychloroquine**	44(42%)	37(34%)

**Prednisolone**	52(50%)	43(41%)

**Cytotoxic drugs**	18(17.3%)	14(13.5%)

**Systemic Lupus Erythematous Disease Activity Index (SLEDAI)**	54(51.92%)	50(48.07)

*Values are means (standard deviation) or absolute numbers (proportions)

*All have no significant differences with non significant *P *value except SLEDAI which is significantly different in two groups (*P *value < 0.004)

We found that QTd was significantly higher in the high-score group than in the low-score group (58.31 ± 18.66 vs. 47.90 ± 17.41 respectively; *P *< 0.004). There was a statistically significant correlation between QTd and cytotoxic medications (azathioprine and cyclophosphamide). In patients who received cytotoxic medications, mean QTd was 61.66 ± 27.77 versus 49.59 ± 15.18 in patients who did not receive cytotoxic medications (*P *= 0.002). Mean QTd in patients who had received hydroxychloroquine was 54.17 ± 19.36 versus 50.82 ± 15.96 in patients who had not received hydroxychloroquine (*P *= 0.45). Corticosteroid treatment was not associated with statistically significant differences in QTd between groups. In patients who had received corticosteroids, mean QTd was 53.58 ± 19.16 versus 50.40 ± 11.59 in patients who had not received corticosteroids (*P *= 0.47). Concerning the effect of azothioprine and cyclophosphamide on QTd we omitted these patients with multiple regression between two groups and new comparison also showed significant difference in QTd between patients with low and high score SLEDAI (54.23 ± 17.32 Vs. 45.90 ± 18.34, *P *= 0.03) (Table 2). There was no correlation between the QTd and baseline characteristics including disease duration, ESR and the presence of any of the diagnostic criteria for SLE. There was no correlation between EF (%), ESV (mL), LV mass (g), IVRT (m/s), IVCT (m/s), tissue Doppler findings in the mitral valve (E, A and S waves), Tie index or SLEDAI (Table 2). However, a significant correlation was found between abnormal echocardiographic findings (including abnormalities in pericardial effusion, pericarditis, pulmonary hypertension and Libman-Sacks endocarditis) and SLEADI. Patients with normal echocardiographic findings had a SLEADI of 11.6 versus 19.13 in patients with abnormal findings (*P *< 0.004).

**Table 2:** Comparison of echocardiographic parameters and QTd between two groups (high score and low score) of SLE patients

	Low score	High score	*P *value
Left ventricular ejection fraction	9.7583 ± .65	11.15 ± 64.32	0.46

ESV (cc)	8.56 ± 25.57	12.02 ± 27.25	0.41

Left ventricular mass(gr)	55.58 ± 160.08	54.11 ± 140.66	0.33

Ewave (cm)	4.72 ± 18.24	4.81 ± 19.94	0.07

Awave (cm)	4.22 ± 14.54	10.16 ± 16.26	0.26

Swave (cm)	3.53 ± 15.30	4.21 ± 15.74	0.56

IVRT (m/sec)*	18.90 ± 77.24	21.10 ± 84.74	0.06

IVCT (m/sec)*	22.23 ± 70.20	22.27 ± 70.59	0.93

Tei index	0.142 ± 0.581	0.218 ± 0.618	0.30

QTd	58.31 ± 18.66 vs. r	47.90 ± 17.41	< 0.004

QTd (in those without use of cyclophosphamide and azothioprine drugs)	54.23 ± 17.32	45.90 ± 18.34	0.03

*- *ESV *End systolic volume, *IVRT *Isovolumic relaxation time, *IVCT *Isovolumic contraction time

## Discussion

Increased QTd has been shown to be an important prognostic factor in several cardiovascular conditions such as coronary artery disease, congestive heart failure and cardiomyopathies [27-31]. Moreover, increased QTd has been documented in several rheumatic diseases [17-19,32] and SLE [20]. To the best of our knowledge, this is the first study to compare QTd in SLE patients with high and low disease activity scores.

Cardiac involvement is the third leading cause of death among patients with SLE. Autopsy studies have documented that the heart is affected in most patients [20]. Pericarditis and premature coronary artery disease are the most common presentations, and other rare associations include myocarditis, coronary arteritis, Libman-Sacks endocarditis, constrictive pericarditis, cardiomyopathy, congenital heart block and rhythm abnormalities [20,32]. Myocardial involvement can be seen in SLE patients even in the absence of clinical cardiac manifestations. Previous studies with Tc-99 m sestamibi myocardial perfusion single-photon emission computed tomography (SPECT) showed a high incidence of myocardial perfusion abnormalities in asymptomatic lupus patients without any clinical signs of cardiac involvement [33-35]. Schillaci et al. found that eight patients with positive SPECT findings had normal epicardial coronary vessels documented with coronary angiography [32]. In patients without clinical cardiac manifestations, these perfusion defects were probably due to the primary immunological damage and small focal cardiofibrosis in this autoimmune disease. The areas of myocardial fibrosis in SLE may disrupt the course of ventricular repolarization and lead to increased dispersion of recovery times throughout the ventricle.

We found that QTd was significantly higher in SLE patients with high disease activity than in patients with mild to moderate disease activity. Because QTd provides useful information on the heterogeneity of ventricular repolarization, increased QTd in the present study may reflect silent myocardial involvement in SLE patients with high activity disease. Therefore, QTd may be a useful simple marker of subclinical myocardial involvement in SLE patients with high disease activity compared to mild to moderate disease activity. The significance of this finding is strengthened by the consideration that hydroxychloroquine, a cytotoxic agent commonly used in SLE patients, cannot in itself explain the increase in QTd. Increased QTd in patients with SLE may be a marker of silent myocardial involvement with its own potentially ominous prognosis. In addition, it can be used as a marker of disease activity or even as a simple way to diagnose SLE itself when other clues are insufficient. Whether increased QTd in patient with SLE predicts poorer clinical outcomes or mandates any special treatments warrants further study.

## Conclusions

QT dispersion was significantly increased in SLE patients with high disease activity compared to patients with low disease activity. Our findings suggest that increased QTd is a potentially useful, simple, noninvasive method for the early detection of subclinical cardiac involvement in patients with high-activity SLE. We suggest that SLE patients with a SLEDAI > 10 may be considered for referral for cardiovascular evaluation.

### Study limitation

Small number of our patients, lack of long term monitoring to detect possible arrhythmia due to long QT dispersion which is an index of arrhythmia susceptibility in high score groups are our study limitations. We did not check anti Ro antibody, and concerning its possible role on prolongation of QT dispersion further study to clarify this effect may be needed.

## Competing interests

Javad Kojuri, Mahammad Ali Nazarinya, Yadollah mahmoodi, Mehdi Ghahartars, Gholamreza Rezaian, Lida Lyaghat have no conflict of interest.

## Authors' contributions

**JK: **main researcher, approving and conducting the reaserch, control of ECG, performing echocardiography, writing the article. **MN: **referring SLE patients, calculating SLEDAI, helping in writing. **MG: **Data gathering, ECG review, preliminary report of article. **YM: **Writing the article, data analysis and statistician coordination. **GRR**: Consultant, QT dispersion calculation and control of quality. **LL**: QTD calculation. All authors read and approved the final manuscript.

## Pre-publication history

The pre-publication history for this paper can be accessed here:

http://www.biomedcentral.com/1471-2261/12/11/prepub
